# Efficient and Specific Generation of *MSTN*-Edited Hu Sheep Using C-CRISPR

**DOI:** 10.3390/genes14061216

**Published:** 2023-06-02

**Authors:** Rihong Guo, Huili Wang, Chunhua Meng, Hongbing Gui, Yinxia Li, Fang Chen, Chenjian Zhang, Han Zhang, Qiang Ding, Jianli Zhang, Jun Zhang, Yong Qian, Jifeng Zhong, Shaoxian Cao

**Affiliations:** 1 Jiangsu Provincial Engineering Research Center for Precision Animal Breeding, Nanjing 210014, China; rhguo@jaas.ac.cn (R.G.);; 2 Institute of Animal Science, Jiangsu Academy of Agricultural Sciences, Nanjing 210014, China; 3 Key Laboratory of Crop and Animal Integrated Farming, Ministry of Agriculture and Rural Affairs, Nanjing 210014, China

**Keywords:** CRISPR/Cas9, Hu sheep, MSTN, double muscling phenotype, off-target effects

## Abstract

Hu sheep, an indigenous breed in China known for its high fecundity, are being studied to improve their growth and carcass traits. MSTN is a negative regulator of muscle development, and its inactivation results in muscularity. The C-CRISPR system, utilizing multiple neighboring sgRNAs targeting a key exon, has been successfully used to generate genes for complete knockout (KO) monkeys and mice in one step. In this study, the C-CRISPR system was used to generate *MSTN*-edited Hu sheep; 70 embryos injected with Cas9 mRNA and four sgRNAs targeting exon 3 of sheep MSTN were transferred to 13 recipients. Out of 10 lambs born from five recipients after full-term pregnancies, nine had complete *MSTN* KO with various mutations. No off-target effects were found. These *MSTN*-KO Hu sheep showed a double-muscled (DM) phenotype, characterized by a higher body weight at 3 and 4 months old, prominent muscular protrusion, clearly visible intermuscular groves, and muscle hypertrophy. The molecular analysis indicated enhanced AKT and suppressed ERK1/2 signaling in the gluteus muscle of the edited Hu sheep. In conclusion, *MSTN* complete KO Hu sheep with a DM phenotype were efficiently and specifically generated using C-CRISPR, and the C-CRISPR method is a promising tool for farm animal breeding.

## 1. Introduction

Hu sheep are a Chinese indigenous breed well known for its high fecundity, early maturity, year-round estrus, high tolerance to heat stress, and suitability for stabling. Hu sheep are introduced to northern areas of China and used as dam lines for commercial breeds. Attempts have been made to cross Hu sheep with meat sheep to breed so-called high-fecundity meat sheep [1], but this is costly and time consuming. Moreover, the cross may introduce undesirable genes and dilute the desired characteristics [2] of Hu sheep. Fortunately, the development of genome editing technology, especially CRISPR (Clustered Regularly Interspaced Short Palindromic Repeats)/Cas9 (CRISPR-associated protein) [3,4], and the identification of myostatin (*MSTN*) as a major gene controlling animal muscularity offer a convenient alternative approach to achieve the breeding goal. 

MSTN belongs to the TGFβ superfamily; it negatively regulates skeletal muscle development [5]. Natural mutations that either inactivate the encoded protein or suppress its quantity cause enhanced muscling in humans [6], sheep [7,8,9], cattle [10,11,12], pigs [13], and dogs [14]. In Texel sheep, an SNP c.*1232G>A in *MSTN* 3′-UTR creating an illegitimate microRNA site reduces 2/3 of MSTN protein level in serum and results in muscularity [8]. In addition to the SNP c.*1232G>A, a frameshift *MSTN* mutation, c.960delG, has been identified in Norwegian white sheep; both SNPs reduced fatness and increased muscle mass in Norwegian white sheep [15]. *MSTN* knockout also results in muscularity in goats [16,17], sheep [18,19,20], cattle [19], and pigs [21,22,23]. Wang et al. [20], Zhou et al. [24], and Li et al. [18] reported that *MSTN* edition in Tan sheep and small tail Han sheep significantly enhanced body weight and muscularity. Consequently, *MSTN* is an ideal target gene to improve the growth and carcass traits of Hu sheep.

CRISPR/Cas9 is an efficient and precision genome editor [3,4] and has been extensively used to generate healthier and more productive farm animals [25,26]. Microinjection of Cas9 mRNA and sgRNA into one-cell-stage embryos generates frameshift mutations at target sites. However, animals generated by this method show gene-functional mosaicism, with gene disruption occurring in some cells but not others [27]. One or two generations are required to produce heterozygous or homozygous edited animals for trait evaluation. However, generation intervals are always long in farm animals, and crossbreeding to generate homozygous individuals is laborious. Using somatic cell nuclear cloning (SCNT) to construct cloning embryos with cells harboring verified mutations can overcome gene-functional mosaicism, and this method has been extensively utilized in farm animal genome editing [18,21,28,29,30,31,32]. Unfortunately, SCNT is hindered by low efficiency and health issues in founder animals. Zuo et al. [33] proposed an alternative method called C-CRISPR, which was successfully used to generate complete gene knockout mice and monkeys in one step. Unlike the standard CRISPR method, C-CRISPR simultaneously uses three to four neighboring gRNAs spaced 10–200 bp apart to target a single key exon of the target gene. This makes C-CRISPR a promising technique for farm animal breeding.

In this study, we used the C-CRISPR method to generate *MSTN*-edited Hu sheep and evaluated the off-target effects. We also analyzed the phenotypic and molecular changes resulting from genome editing.

## 2. Materials and Methods

### 2.1. Animals

The Hu sheep were maintained at the Luhe Animal Scientific Base of the Jiangsu Academy of Agricultural Sciences in Jiangsu province. The experimental procedures were approved by the Research Committee of the Jiangsu Academy of Agricultural Sciences and conducted with adherence to the Regulations for the Administration of Affairs Concerning Experimental Animals (Decree No. 63 of the Jiangsu Academy of Agricultural Science on 8 July 2014).

### 2.2. Preparation of Cas9 mRNA and sgRNA

The sgRNA design and in vitro transcription (IVT) of Cas9 mRNA and sgRNA were described in our previous study [17]. Briefly, four sgRNAs (Supplementary Table S1) targeting sheep *MSTN* exon 3 were designed with CRISPR tools (https://benchling.com/, accessed on 15 September 2020). The oligos for each sgRNA (Supplementary Table S1) were annealed and cloned into the pX330 plasmid. The IVT templates for Cas9 and sgRNAs were amplified using the T7 promotor-appended primers (Supplementary Table S2) and were gel-purified using a QiaQuick Spin Column (28104Qiagen, Shanghai, China). The Cas9 IVT template (400 ng for a 20 µL reaction) was subjected to a T7 Ultra Kit (AM1345, Ambion, Shanghai, China), and sgRNA IVT templates (200 ng for a 20 µL reaction) were transcribed using the MEGAshortscript Kit (AM1354, Ambion) in vitro. The Cas9 mRNA and sgRNAs were purified using the MEGAclear Kit (AM1908, Ambion). The purified RNAs were quantified using NanoDrop 2000 and then subjected to electrophoresis in 1.5% agarose gel (Supplementary Figure S1). The high-quality RNAs were stored at −80 °C before use.

### 2.3. Manipulation of Sheep Embryos

Sheep superovulation and estrous synchronization, embryo collection, injection, and transfer were performed as previously described [17] with modifications (Supplementary Figure S2). In brief, healthy donors (*n* = 5) and recipients (*n* = 13) with normal estrous cycles were intravaginally implanted with progesterone sponges (EAZI-BREED CIDR Sheep and Goat Devices, ZOETIS, Rhodes, NSW, Australia) for 13 days, followed by administration of 0.1 mg PGF2α analogues (Cloprostenol Sodium Injection) at the time of sponge removal. The donors received 675 IU FSH twice daily in a decreasing dose over 3.5 days (200/150, 100/75, 75/50, and 25 IU) starting 72 h before sponge removal. Then donors were naturally mated twice at 36 and 48 h after sponge removal. The recipients received 400 IU PMSG 72 h before sponge removal. All hormones or analogues used in this study were provided by Sansheng Pharmaceutics (Ningbo, China).

Twenty hours after the last insemination, the one-cell-stage embryos were flushed from the oviducts, and the collected zygotes were injected with a mixture of Cas9 mRNA (100 ng/µL) and sgRNAs (50 ng/µL for each sgRNA). The injected zygotes were cultured in M2 media containing 10% FBS (26140, Gibco, Shanghai, China) at 37 °C for 2 to 4 h, and then live embryos were transferred to estrous-synchronized recipient sheep.

### 2.4. Detection of Genome Editing at the Target Sites and POTSs

Blood or ear tissue genomic DNA of the newborn lambs was extracted using a DNA extraction kit (DP348, Tiangen, Beijing, China). The genomic regions surrounding exon 3 were amplified by PCR using the primers listed in Supplementary Table S3. The purified PCR amplicons were subjected to Sanger sequencing and subcloned into the pMD-19T vector (Takara, D103A), with 10–20 colonies randomly selected from each sample for sequencing. 

The potential off-target sites (POTSs) of sgC1-C4 were computationally predicted using Cas-OFFinder according to the Hu sheep genome assembly GCA_011170295.1_ASM1117029v1. The POTS sites were PCR amplified and subjected to Sanger sequencing. The POTSs and primers used for the amplification of the target sites and POTSs are listed in Supplementary Table S4.

### 2.5. Western Blotting and Histological Analysis

Gluteus muscle biopsies were surgically collected from a control and an *MSTN*-edited Hu sheep (#521) 12 months of age. The aliquots of the muscle biopsies were either fixed by 4% paraformaldehyde for hematoxylin and eosin (HE) staining or immediately immersed into liquid nitrogen for storage. 

For the Western blotting analysis, the total protein of the frozen muscle sample was extracted using RIPA lysis buffer (P0013B, Beyotime Biotechnology, Nantong, China) with phosphatase inhibitor cocktail C (P1091, Beyotime Biotechnology). Then, the lysates were boiled in the gel-loading buffer, and 30 µg of protein was separated by SDS-PAGE in each lane of a 12% gel. The proteins were subsequently transferred to a polyvinylidene fluoride membrane (Millipore, Darmstadt, Germany) and probed with primary antibodies against GAPDH (60004-1-Ig, Proteintech, Wuhan, China), MSTN (19142-1-AP, Proteintech), ERK1/2 (4695T, Cell Signaling Technology, Danvers, MA, USA), p-ERK1/2 (4370T, Cell Signaling Technology), AKT (9272S, Cell Signaling Technology), p-AKT (4060S, Cell Signaling Technology), P38 (9272S, Cell Signaling Technology), and p-P38 (8690S, Cell Signaling Technology). The chemiluminescence was detected by an ECL kit from Pierce Chemical (Dallas, TX, USA) and visualized through Image Quant LAS 4000 (Fujifilm, Tokyo, Japan). The band intensity was quantified with ImageJ software (NIH, Bethesda, MA, USA).

For the histological analysis, the fixed muscle samples were dehydrated in a graded alcohol series and then embedded in paraffin. The paraffin-embedded tissues were sectioned at 3–5 µm. The slices were then stained with an HE kits (C0105S, Beyotime Biotechnology). The stained slides were viewed via microscopy (Leica, Wetzlar, Germany). The myofiber sizes from five randomly selected fields of view in each slice were analyzed using the Image Pro Plus software (Media Cybernetics, Silver Spring, MD, USA). 

### 2.6. Data Analysis

Data were presented as mean ± SEM. The differences in body weight and myofiber size were analyzed using t-tests. Statistical analyses were performed using GraphPad Prism (Version 9.0). A *p*-value < 0.05 was considered significant.

## 3. Results

### 3.1. Efficient Generation of MSTN-Edited Hu Sheep Using C-CRISPR

**Figure 1:**
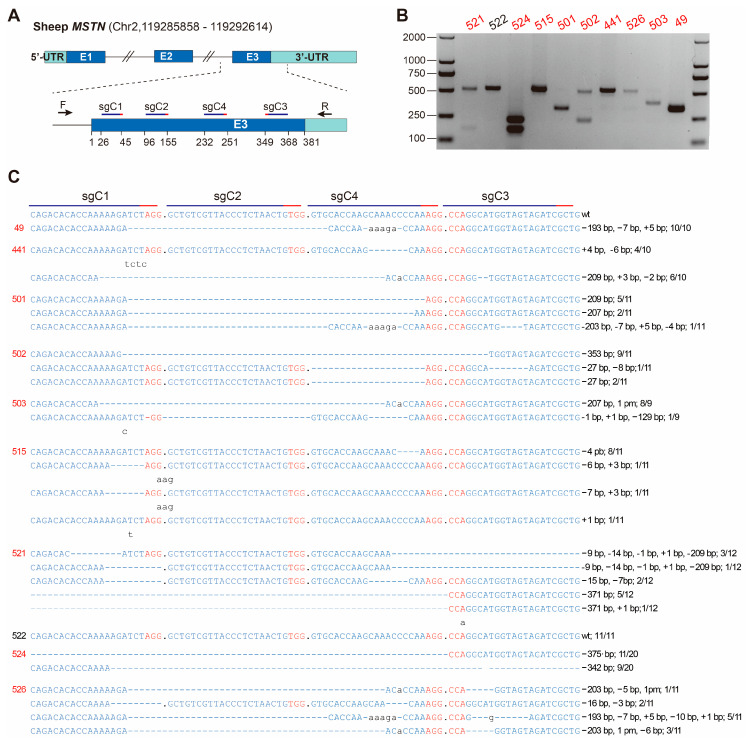
Generation of *MSTN*-edited Hu sheep using C-CRISPR. (**A**) Four sgRNAs (sgC1–C4) targeting sheep *MSTN* exon 3 were designed, and the numbers below the panel indicate the position in the 381 bp exon 3. F/R were primers used for amplification of exon3. (**B**) The target site amplification result of the ten newborn lambs. The nine edited lambs are highlighted in red. (**C**) TA clone sequencing results. The mutated and inserted bases are indicated in lowercase, and the deletions are indicated by “−”. WT: wild type; −: deletion; +: insertion; N/N indicates the positive colonies of the total colonies sequenced.

Four sgRNAs (sgC1–C4) targeting the sheep *MSTN* exon 3 were designed (Figure 1A, Supplementary Table S1). The efficiencies of the sgRNAs were not verified in cells or in vitro cultured embryos. A total of 83 embryos were collected from five donors and injected with a mixture of Cas9 mRNA, sgC1–C4. After culturing for 1–2 h, 70 live embryos were transferred to 13 recipients. Five recipients delivered 10 lambs after full-term pregnancies. (Table 1).

Sanger sequencing of the target sites showed that 9 out of the 10 lambs were edited (Figure 1B,C). The TA clone sequencing results indicated that the mutation ratios in all the edited sheep were 100%, and each lamb had one to five types of indels, which were −371 bp ~ +1 bp. Indels at sgC1 target sites were present in all nine edited lambs, and all mutations harbored indels at sgC1 sites except lamb #515, whose indel ratio at sgC1 sites was 27.27%. For sgC4, eight out of the nine lambs had indels and were 100% edited except lamb #515 (the mutation rate at sgC4 sites was 72.72%). Five lambs had indels at sgC3 and none at sgC2. The indel ratio at the sgC3 target site varied from 9.09% to 100%. By combining the indels of sgC1 and sgC4, *MSTN* in eight out of the nine lambs was 100% edited. By combining the indels of sgC1, sgC3, and sgC4, *MSTN* in the nine lambs was 100% edited. Notably, there were long-range deletions across sgC1 and sgC4 (~190 bp) and sgC4 and sgC3 (~209 bp), as well as sgC1 and sgC3 (~353 bp), with the most frequent type of long-range deletion across sgC1 and sgC4 in eight out of the nine edited lambs.

### 3.2. Specificity of Genome Editing Using C-CRISPR

To evaluate the off-target effects, potential off-target sites (POTSs) with PAM NGG of these four sgRNAs were predicted using Cas-OFFinder [34] according to the reference genome. The details of these POTSs are listed in Supplementary Table S4, with six to seven POTSs detected for each sgRNA. No de novo mutations were found among these POTSs after PCR and Sanger sequencing. Multiple alignments of the Sanger sequencing results are presented in Supplementary Figure S3.

### 3.3. Phenotype Analysis of MSTN-Edited Hu Sheep

**Figure 2:**
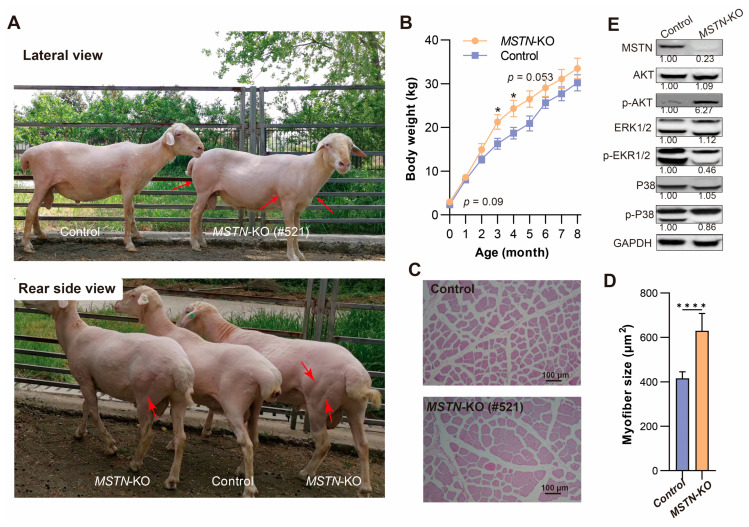
Phenotype and molecular analysis of *MSTN*-edited Hu sheep. (**A**) Pictures of *MSTN*-KO and control male sheep. The top and bottom pictures were taken from the lateral view and the rear side view, respectively. Red arrows indicate muscular protrusion, intermuscular boundaries, and grooves in the forequarters and hindquarters of *MSTN*-KO sheep. (**B**) Growth curve of *MSTN*-KO and control Hu sheep from birth to 8 months. Shown are mean values ± SEM. * *p* < 0.05. (**C**,**D**) HE-stained cross section and myofiber size of gluteus muscles from *MSTN*-KO (#521) and control sheep. **** *p* < 0.001. (**E**) Western blot analysis of MSTN, AKT, p-AKT, ERK1/2, p-EKR1/2, p38, p-P38 and GAPDH. The number under the blot picture is the relative protein expression relative to the control group.

*MSTN*-KO Hu sheep displayed double-muscled (DM) phenotype (Figure 2A). This was particularly evident in the proximal regions of both the forequarters and hindquarters, where there was a prominent muscular protrusion. The intermuscular boundaries and grooves were also clearly visible beneath the skin.

The body weights of the edited lambs (*n* = 9) and the control lambs (*n* = 8) were monitored from birth to 8 months old (Figure 2B). At birth, the edited lambs showed a trend toward higher body weights compared with the control group (*p* = 0.09). This difference became significant at 3 and 4 months old. However, as the lambs grew older, the difference in body weight between the two groups gradually diminished and was no longer significant from 5 months old (*p* = 0.053). 

The histological analysis of the gluteus muscle indicated muscle hypertrophy (Figure 2C). The cross-sectional area of the muscle fibers in the edited Hu sheep increased 51.4% compared with the control Hu sheep (630.00 ± 78.56 vs. 416 ± 35.19 µm^2^, *p* < 0.001) (Figure 2D). 

### 3.4. Molecular Analysis of MSTN-Edited Hu Sheep

Constant with the genome editing at the *MSTN* locus, mature MSTN protein of the gluteus muscle decreased 77% in the edited Hu sheep compared with the control Hu sheep (Figure 2E). The remaining MSTN protein in edited sheep had a slightly lower molecular weight, indicating truncation of the MSTN protein. Among the downstream signaling mediators of MSTN (AKT, ERK1/2, and P38), p-AKT increased by 6.27-fold, while p-ERK1/2 decreased by 0.46-fold. p-P38 remained unchanged (0.86-fold) (Figure 2E). These results indicated that the activation of AKT signaling and repression of ERK1/2 signaling contributed to muscle hypertrophy in the *MSTN*-KO Hu sheep.

## 4. Discussion

In China, intense cross-breeding efforts have been afforded to improve the carcass performance of Hu sheep. However, the grading-up process is labor intensive and time consuming, and other desired genes may be diluted. In this study, we successfully achieved complete *MSTN* gene KO in 90% of newborn lambs using the C-CRISPR method. This high level of gene KO efficiency has never been reported before in genome editing of farm animals. Furthermore, the *MSTN*-KO Hu sheep founders exhibited the typical DM phenotype, with obvious intermuscular boundaries and grooves in both forequarters and hindquarters. These results indicate that the C-CRISPR method is a promising tool for breeding farm animals.

The C-CRISPR method was first proposed by [33]; they generated gene total KO founder animals with adjacent sgRNAs in one step. The high efficiencies of C-CRISPR can be easily understood. As the efficiency of each sgRNA is less than 100%, the gene editing must result in gene function mosaicisms when one sgRNA is used. For example, in our *MSTN*-edited Haimen goats and rabbits and *CLPG1*-edited rabbits, the efficiencies of the three sgRNAs targeting *MSTN* were from 11.1 to 100% [17], and the efficiencies of the two sgRNAs targeting *CLPG1* were from 20.0 to 100% [35]. In Wang et al. [20], five sgRNAs were used to edit the sheep genome; the efficiencies of these sgRNAs were as follows: *MSTN* sg1, 4.3 to 100%; *MSTN* sg2, 16.6 to 100%; *ASIP* sg1 + sg2, 11.1 to 60%; and *BCO2* sg1 + sg2, 9.5% to 100%. When two or more sgRNAs are used, the total genome editing efficiency can be calculated as *p* = 1 − (1 − *p*_1_) (1 − *p*_2_)…(1 − *p*_N_), while *P*_1_, *P*_2_… *P*_N_ are the genome editing efficiencies of the each sgRNA, and N is the number of sgRNAs used in C-CRISPR. The *p* value will gradually increase to 100% as the number of sgRNAs increases and as the values of *p*_1_, *p*_2_… *p*_N_ increase. The present study achieved total gene knockout mediated by two (sgC1 and sgC4) and three sgRNAs (sgC1, sgC3, and sgC4). These results support that C-CRISPR is efficient, even with sgRNAs whose efficiencies have not been validated in advance, and it could generate genetic function homozygous KO farm animals in one step.

CRISPR/Cas9 has been proven to be high fidelity in several in vivo studies [36,37,38] and stem cell research [39,40,41] using whole-genome sequencing; rare but bona fide off-target mutations were reported in founder mice [42,43]. In our current study, no de novo mutations were found in the founders. The high fidelity of our genome editing in Hu sheep is in line with Wang et al. [20] and Wang et al. [44], who proved the genome editing specificity in sheep by genotyping POTSs predicted in silico [20] or by whole-genome sequencing [44]. 

Our *MSTN*-KO Hu sheep founders exhibited the DM phenotype characterized by prominent higher body weight, muscular protrusion, clearly visible intermuscular groves, and muscle hypertrophy. This was consistent with the muscularity in other sheep with *MSTN* natural mutations [8,9,15,45] or *MSTN* KO [18,20,46]. The average fiber size increased 51.4% in our *MSTN*-KO Hu sheep, and the muscle hypertrophy was consistent with [18,24], which may contribute to their muscularity. The body weight of the edited Hu sheep significantly increased at 3 and 4 months and tended to be higher at 0 d or 5 months, which was in line with the effects of *MSTN* knockout on sheep growth performance. The enhancement effects of *MSTN* knockout on body weight vary in the literature. Crispo et al. [46] generated homozygous and heterozygous *MSTN*-edited sheep guided by sgRNA targeting exon 1 and found that the body weight of homozygous *MSTN* knockout sheep was significantly higher from 15 d to 30 d old and tended to be higher at 60 d old. Wang et al. [20] generated *MSTN*-knockout Tan sheep by microinjection of Cas9 mRNA and two sgRNAs targeting exon 2 and exon 3, the efficiencies ranging from 4.34% to 100%, and the body weight of the edited sheep was higher at birth, 1 month old, and 5 months old. Li et al. [18] generated *MSTN*-edited small tail Han sheep using TALEN by targeting exon 1 of ovine *MSTN*, and the body weights were significantly higher from 1 to 7 months old. 

The effects of *MSTN* edition on phenotype are target location, breed, and editing efficiency dependent. First, *MSTN* editing has a target location effect [47]. For example, all *MSTN*^−/−^ large white pigs with exon 3 mutations showed lameness and only survived for 1–2 days, while *MSTN*^−/−^ piglets with exon 1 mutations were healthy [23]. Additionally, the well-known sheep *MSTN* mutations c.*1232G>A and c.960delG lead to the typical double muscle phenotype and exist in Norwegian white sheep. However, after 30 years of breeding for improved carcass quality, only the frequencies of c.*1232G>A increased [7]. This is because most homozygous c.960delG lambs die after birth for unknown reasons [7], while homozygous c.*1232G>A sheep are healthy. Second, the effects of *MSTN* mutation are breed dependent. As mentioned above, *MSTN*^−/−^ large white pigs are born with health problems, while *MSTN*^−/−^ Meishan pigs with mutations at exon 3 are healthy. Last, the effects of MSTN are dosage dependent; the genome editing efficiencies are key factors influencing the growth performance. Thus, the differences in sgRNA used (different target sites), the sheep breed, and also editing efficiencies may all contribute to the variation in body weight and other phenotypes between studies.

MSTN functions as a negative regulator of muscle development [5] and satellite cell proliferation and differentiation [48,49]. The mechanisms are not fully understood, but it is known that MSTN acts through several downstream mediators including the TGFβ signaling pathway, TGFβ signaling pathway [5,50], AKT [51,52,53], P38 [54], and ERK1/2 [55]. In in vitro cultured myoblasts, the repression of AKT signaling was involved in MSTN-inhibited, both in differentiation in myoblasts and hypertrophy in myotubes [52,53]; in vivo overexpression of *MSTN* resulted in muscle loss through inhibiting AKT signaling [49]. In contrast, AKT signaling was enhanced in myostatin-deficient mice [56] and pigs [29]. Our results also showed a 6.27-fold increase in AKT activation. The ERK1/2 cascade has been reported to be activated by myostatin in C2C12 myoblasts and blocking ERK1/2 activation attenuates MSTN-suppressed myotube fusion and differentiation [55]. ERK1/2 has also been reported to be involved in MSTN-suppressed progesterone secretion in ovary granulosa cells [57] and preadipocyte differentiation [58,59]. In our study, we found that ERK1/2 activation decreased by 0.57-fold. P38 has also been shown to be activated by MSTN in C2C12 myoblasts, and treatment with P38 inhibitors reduces the MSTN-induced inhibition of proliferation [54]. However, P38 was only slightly changed in our study. Our results emphasize the importance of AKT and ERK1/2 signaling in MSTN-regulated muscle function in Hu sheep.

Recently, PRLR knockout SLICK cattle were approved by the FDA as a human food source after strict molecular characterization and assessment of the phenotypic data and animal safety, human food safety, and environmental risks [60]. This has paved the way to utilize genome editors as domestic animal precision breeding tools. Accordingly, *MSTN*-edited Hu sheep have the potential to be approved as a human food source after molecular characterization and evaluation of phenotypic data, animal safety, human food safety, and environmental risks. In the future, other phenotypic data such as carcass traits and meat traits will be evaluated after preparing enough offspring, along with an assessment of the environmental risks.

In conclusion, our study indicated that C-CRISPR is a promising method in farm animal breeding. In addition, *MSTN*-edited Hu sheep with the DM phenotype were efficiently and precisely generated.

## Figures and Tables

**Table 1:** Summary of the generation of *MSTN*-edited sheep using C-CRISPR.

sgRNAs	Donors Superovulated	Embryo Injected/Embryos Transferred	Delivered Recipients /Total Recipients	Edited Lambs /Total Lambs
sgC1–C4	5	83/70	5/13 (38.46%)	9/10 (90.00%)

## Data Availability

The data presented in this study are available on request from the corresponding author.

## Supplementary Materials

The following supporting information can be downloaded at: https://www.mdpi.com/article/10.3390/genes14061216/s1, Supplemental Figure S1. Electrophoresis results of IVT templates and RNAs for Cas9 and sgRNAs. Supplemental Figure S2. Protocols for superovulation and estrous synchronization of Hu sheep. Supplemental Figure S3. Detection of the off-target effects by Sanger sequencing of the POTS for sgC1, sgC2, sgC3, and sgC4. Supplemental Table S1. Information of sgRNA and oligos for sgRNA construction. Supplemental Table S2. Primers for IVT template amplification. Supplemental Table S3. Primers for exon3 amplification. Supplemental Table S4. Information on the potential off-target sites (POTs) for sgC1-C4 and the primers for PCR amplification of the sites.
