# Supplementary material for: Efficient and Specific Generation of MSTN-Edited Hu Sheep Using C-CRISPR
Source: Genes (Basel). 2023 Jun 2;14(6):1216. doi: 10.3390/genes14061216 (PMC10298037; doi:10.3390/genes14061216)
Supplement: Supplementary file 1 [file genes-14-01216-s001.zip › genes-2333673-supplementary.pdf]

Supplemental materials

List of the Supplemental figures and tables:

Supplemental Figure S1. Electrophoresis results of IVT templates and RNAs for Cas9 and sgRNAs.

Supplemental Figure S2. Protocols for superovulation and estrous synchronization of Hu sheep.

Supplemental Figure S3. Detection of the off-target effects by Sanger sequencing of the POTS for sgC1, sgC2, sgC3, and sgC4.

Supplemental Table S1. Information of sgRNA and oligos for sgRNA construction.

Supplemental Table S2. Primers for IVT template amplification.

Supplemental Table S3. Primers for exon3 amplification.

Supplemental Table S4. Information on the potential off-target sites (POTs) for sgC1-C4 and the primers for PCR amplification of the sites.

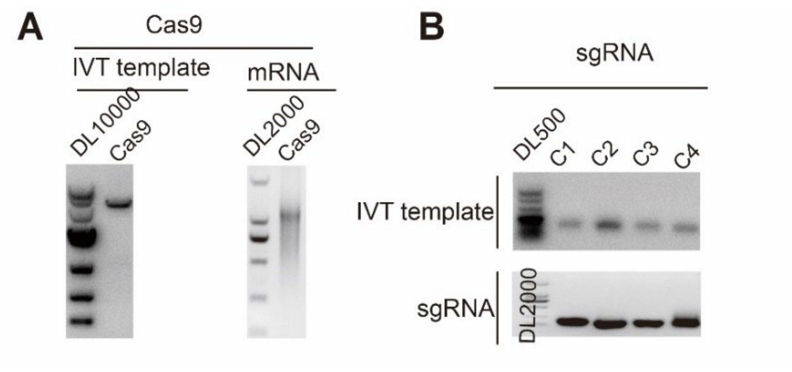

Supplemental Figure S1. Electrophoresis results of IVT templates and RNAs for Cas9 (A) and sgRNAs (B).

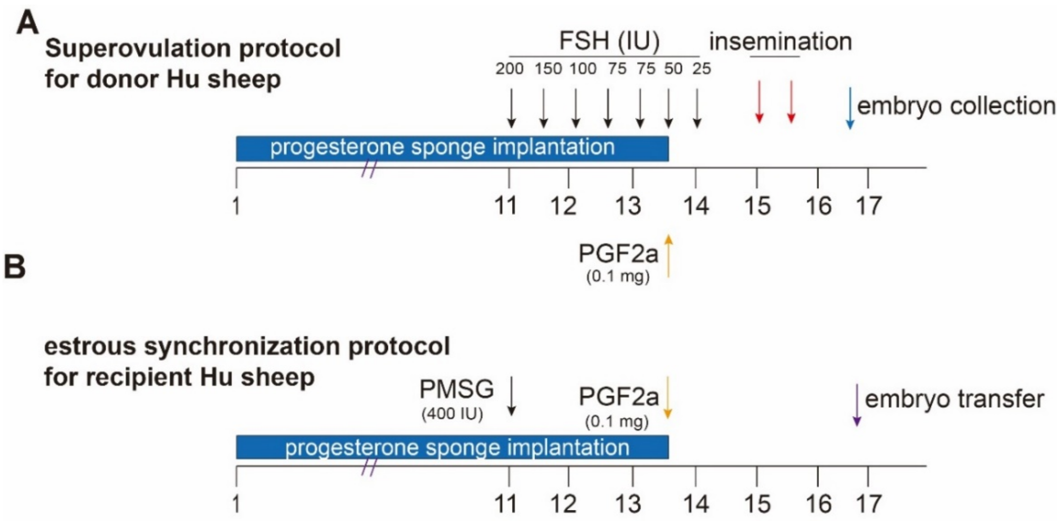

Supplemental Figure S2. Protocols for superovulation (A) and estrous synchronization (B) of Hu sheep.

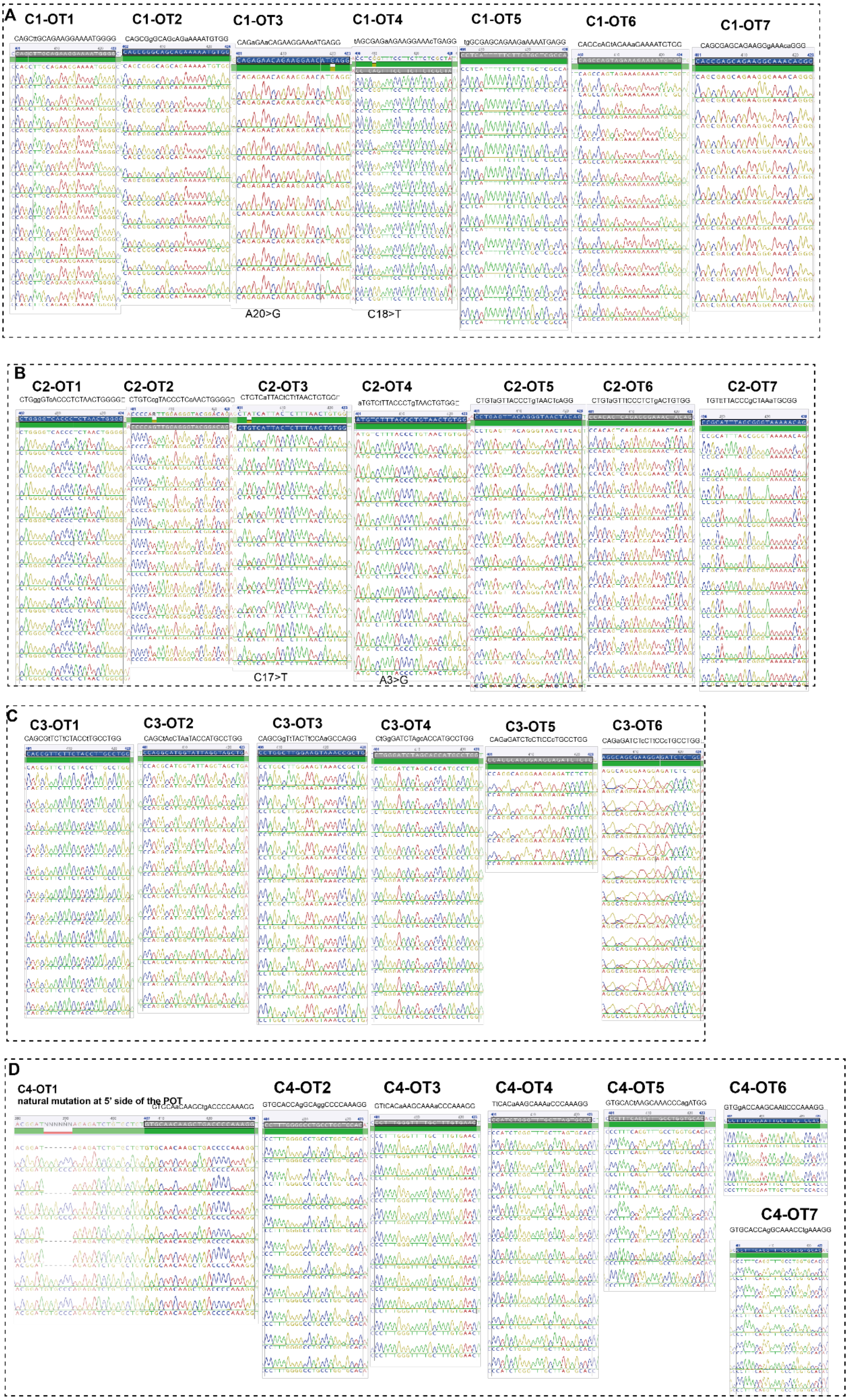

Supplemental Figure S3. Detection of the off-target effects by Sanger sequencing of the POTS for sgC1 (A), sgC2 (B), sgC3 (C), and sgC4 (D).

Supplemental Table S1. Information of sgRNA and oligos for sgRNA construction

sgRNA	sgRNA sequences	primers for sgRNA construction
sgC1	CAGACACACCAAAAAGATCT	F: caccgCAGACACACCAAAAAGATCT<br>R: aaacAGATCTTTTTGGTGTGTCTGc
sgC2	CTGTCGTTACCCTCTAACTG	F: cacc CTGTCGTTACCCTCTAACTG<br>R :aaacCAGTTAGAGGGTAACGACAGc
sgC3	CAGCGATCTACTACCATGCC	F: caccgCAGCGATCTACTACCATGCC<br>R: aaacGGCATGGTAGTAGATCGCTGc
sgC4	GTGCACCAAGCAAACCCCAA	F: caccGTGCACCAAGCAAACCCCAA<br>R: aaacTTGGGGTTTGCTTGGTGCAC

Supplemental Table S2. Primers for IVT template amplification

IVT_sgC1_F	TtaatacgactcactataggCAGACACACCAAAAAGATCT
IVT_ sgC2_F	TtaatacgactcactataggCTGTCGTTACCCTCTAACTG
IVT_ sgC3_F	TtaatacgactcactataggCAGCGATCTACTACCATGCC
IVT_ sgC4_F	TtaatacgactcactatagGTGCACCAAGCAAACCCCAA
IVT_sgRNA_R	AAAAGCACCGACTCGGTGCC
IVT_Cas9_F	taatacgactcactatagggAGAATGGACTATAAGGACCACGAC
IVT_Cas9_R	GCGAGCTCTAGGAATTCTTAC

Supplemental Table S3. Primers for exon3 amplification

Primers	Primer sequences
Ovis_exon3_F	ACTGAGGAAAACAGCGATAAACA
Ovis_exon3_R	GCACCCACAGCGATCTACTA

Supplemental Table S4. Information on the Potential off-target sites (POTs) for sgC1-C4 and the primer for PCR amplification of the sites.

	POTs	chromosome	location	Direction	No. mismatches bases	Primers	Product length
C1	CAGCGAGCAGAAGGAAAATGTGG	2	129059831	+	0		
C1_OT1	CAGCttGCAGAAGGAAAATGGGG	3	186543474	+	2	F: AGCCTGCTAGCTTGCTGAAA<br>R: GCCCACCACCCAGATAAGAC	522
C1_OT2	CAGCGgGCAGcAGaAAAATGTGG	2	52592129	+	3	F: CTCAGCAGGGTCTTTCCCAG<br>R: TGTTCCTGGATGCTGCATGT	494
C1_OT3	CAGaGAaCAGAAGGAAcATGAGG	2	170571182	+	3	F: AAAAACTGGAGGCCACAGGG<br>R: GGCCCAGGCTCACATTTACT	409
C1_OT4	tAGCGAGaAGAAGGAAAcTGAGG	3	21201734	-	3	F: GGGAGGGGAGAGTATGGGTT<br>R: ACTCAGGAAGTGTCTGCTGC	538
C1_OT5	tgGCGAGCAGAAGaAAAATGAGG	3	61816130	-	3	F: CAAGCTCGAGAGTCAGTGGG<br>R: GCTGGATCAGCATGAGGGTT	411
C1_OT6	CAGCcAGtAGAAaGAAAATGTGG	4	101638513	+	3	F: TGCTGGAGTTGAACCCTTCC<br>R: ATCTCGTGAGACGAGGGTCA	472
C1_OT7	CAGCGAGCAGAAGGgAAAcaGGG	5	64472003	+	3	F: GATGGTGGCATTCACTGGGA<br>R: CAGGACCTGCATCCTCTTCC	525
C2	CTGTCGTTACCCTCTAACTGTGG	2	129064459	+	0		
C2_OT1	CTGggGTcACCCTCTAACTGGGG	1	2654177	+	3	F: TTCATCCTGAGGCTGCAGTG<br>R: GAGGCCTGGCTGTATCTGTC	568
C2_OT2	CTGTCcgTACCCTCcAACTGGGG	2	74578208	-	3	F: CTGTACCGGATCCCATCAGC<br>R: GATACACTGCTTTGCGCTCG	528
C2_OT3	CTGTCaTTACtCTtTAACTGTGG	4	120785424	+	3	F: TATGCTTGTCAGGGTGGATGC<br>R: GCGAACCCTCAGAACCTACT	479

C2_OT4	aTGTCtTTACCCTgTAACTGTGG	10	18051994	+	3	F: GAGGTGGAGTGTTCTGGAGC<br>R: GCTTCAGGCACAGGATGAGT	429
C2_OT5	CTGTaGTTACCCTgTAACTcAGG	20	46890723	-	3	F: TCCTACCATCGTGTAATGCCA<br>R: GCTTTCTTGATTACACAAAAGGACA	430
C2_OT6	CTGTaGTTtCCCTCTgACTGTGG	23	28180972	-	3	F: TTCACTGAACTAGCTGCGGG<br>R: CCATTGCATCCTTGACCCCT	418
C2_OT7	CTGTttTTACCCgCTAAaTGCGG	1	190085274	-	4	F: CTGCCCAGCATGTGTTAAATAGT<br>R: GCCCATCATATGGACATTGGG	626
C3	CAGCGATCTACTACCATGCCTGG	2	129064709	-	0		
C3_OT1	CAGCGtTCTtCTACCtTGCCTGG	23	23035138	+	3	F: AGGCAAGCCTGGGTACTCTA<br>R: TCATGGCCATCAACCTGGAC	709
C3_OT2	CAGCtAcCTAaTACCATGCCTGG	24	6772888	-	3	F: ACCAATCTCCAAACCCTAATGC<br>R: CCTGGGCGTACTAGCAATCAA	495
C3_OT3	CAGCGgTtTACTtCCAaGCCAGG	1	156763025	-	4	F: CTGAAACTATGGTCAGGCAGC<br>R: TGAGGGGAAAGAAGGGTTACCA	428
C3_OT4	CtGgGATCTAgcACCATGCCTGG	2	205510233	+	4	F: GTTGGTTGTCTGCAGTCTTGAA<br>R: AGGAATAAGGGTGGCAGTCATT	449
C3_OT5	CAGaGATCTcCTtCCcTGCCTGG	3	180798205	-	4	F: GCTCTCAGTCACCACAGCTT<br>R: TGCAGCAGTGAGCAGATTCA	454
C3_OT6	CAGaGATCTcCTtCCcTGCCTGG	3	181226657	-	4	F: TTGATGCAGCTTCCGTCTGT<br>R: CTCAGTGGCTCAGCTGTGAA	304
C4	GTGCACCAAGCAAACCCCAAAGG	2	129064595	+	0		
C4_OT1	GTGCAaCAAGCtgACCCCAAAGG	2	112771869	+	3	F: AAAACGGAAGCAAGCCTCCTAC<br>R: TCTCCTCAAATCCTGGCAACCA	260

C4_OT2	GTGCACCAgGCAggCCCCAAAGG	5	68695419	-	3	F: CACAGTGTCCAAGTTGGGGT<br>R: TTCACCAGCGACCTGACTTC	407
C4_OT3	GTtCACaAAGCAAAaCCCAAAGG	6	112618982	-	3	F: CTCCTGCCCACAACAAATTCT<br>R: CAGACCTAAGTGGCGGTAAAATTC	734
C4_OT4	GTtCACaAAGCAAAaCCCAAAGG	6	112641342	-	3	F: GGCACGAATGGAGCTCATGTA<br>R: TGTCAGACCTAAGTGGCGGTAA	507
C4_OT5	GTGCACtAAGCAAACCCagATGG	18	14602839	-	3	F: GTGTGGATTTCTCAAGCGCA<br>R: GGCCTAGTTAAGGAAGGGCA	445
C4_OT6	GTGgACCAAGCAAttCCCAAAGG	20	15935692	-	3	F: ATTCTGTGCAGCCAGTCTCC<br>R: AGCTTGGTGCGTGTGGATTA	727
C4_OT7	GTGCACCAgGCAAACCtgAAAGG	23	48830102	-	3	F: GATGGGGAGGGGTTGTGAAG<br>R: ACCCTGTCTCCCACATCGTA	277
